# Measurement of diffusion in articular cartilage using fluorescence correlation spectroscopy

**DOI:** 10.1186/1472-6750-11-19

**Published:** 2011-03-02

**Authors:** Jeong Ik Lee, Masato Sato, Kiminori Ushida, Joji Mochida

**Affiliations:** 1 Department of Orthopaedic Surgery, Surgical Science, Tokai University School of Medicine, 143 Shimokasuya, Isehara, Kanagawa 259-1193, Japan; 2 Department of Biomedical Science & Technology, Institute of Biomedical Science & Technology (IBST), Konkuk University, 1 Hwayang-dong, Gwangjin-gu, Seoul 143-701, Korea; 3 Eco-Soft Materials Research Unit, RIKEN (The Institute of Physical and Chemical Research), 2-1 Hirosawa, Wako, Saitama 351-0198, Japan

## Abstract

**Background:**

Fluorescence correlation spectroscopy (FCS) provides information about translational diffusion of fluorescent molecules in tiny detection volumes at the single-molecule level. In normal states, cartilage tissue lacks vascularity, so chondrocyte metabolism depends on diffusion for molecular exchanges. The abundant extracellular matrix (ECM) of cartilage is maintained by a limited number of chondrocytes. ECM plays an important role in the regulation of chondrocyte functions. In this study, FCS was used to measure diffusion behaviors of albumin, the major protein of the intra-articular space, using normal and degenerated cartilage. Preliminary investigation of fluorescence dyes including Alexa 488, Rhodamine 6G and Rhodamine 123 was conducted to evaluate their properties in cartilage.

**Results:**

The results indicate that the diffusion behaviors of fluorescently lableded albumin can be observed using FCS in both normal and chemically degenerated cartilage.

**Conclusions:**

This work demonstrates the capability of FCS for direct measurement of diffusion in cartilaginous ECM. When the diffusion characteristics of fluorescent probes in ECM are clarified using FCS evaluation, FCS will be applicable as a method for early diagnosis of osteoarthritis, which is accompanied by increased abnormalities of ECM and also as tool for evaluating bio-engineered artificial cartilage for autologous chondrocyte implantation.

## Background

Fluorescence correlation spectroscopy (FCS) is a highly sensitive method based on analysis of fluctuations in fluorescence intensity to detect and characterize fluorophores in living cells as well as in solution. For instance, FCS allows real-time measurement of two important physical parameters for biochemistry: the average number of molecules in the detection space; and the translational diffusion constant of the molecules through the open volume of detection [1-4].

Cartilage tissue is an avascular tissue, and allows the exchange and transport of nutrients, gases, and metabolites by continuous diffusion instead of through the vasculature [5]. Diffusion in extracellular matrix (ECM) of normal cartilage is thus central to the physiobiological nature of chondrocytes. Cartilage tissue principally consists of ECM and a small number of chondrocytes. The abundant ECM in cartilage is secreted by these chondrocytes. Although ECM provides an environment for the molecular exchanges needed for chondrocyte survival, and plays an important role in physiological activities for the regulation of chondrocyte function, intimate communications between cells and alterations of metabolism, almost no studies have examined the diffusion behaviors of particular molecules from synovial fluid through the ECM of cartilage. Some studies have examined diffusion characteristics and diffusion across articular cartilage using dyes [6,7], glucose [6] and hydrogen [8], To investigate the normal pattern of every different type of molecules in ECM of cartilage, large-scale experiments and varying samples are required. These efforts may help define intricate phenomenon of diffusion in cartiliginous tissue.

Since synovial fluid makes a significant contribution to the nutrition of articular cartilage with direct movement of particular molecules from the synovial space to cartilage by diffusion, understanding the diffusion patterns of molecules from synovial fluid is important. Pathological changes to the ECM cause osteoarthritis (OA), altering not only the physical metabolism of chondrocytes, but also normal molecular exchanges in cartilage.

In this study, FCS analysis is made to evaluate albumin movements by means of diffusion in cartilage. Since albumin is the major protein of synovial fluid, tracing albumin protein movements may reveal differences between normal and abnormal states of cartilage.

The purpose of this study was to evaluate the feasibility of FCS for diffusional analysis in normal and chemically degenerated cartilage in relation to albumin, as a representative protein. To select a suitable fluorescent dye before application to cartilage tissue, the physical parameters of several fluorescent dyes were tested and documented. We chose to use a model of degenerated cartilage created by chemical treatment for FCS evaluation, to reflect the denaturation of ECM that might be expected in cartilage tissue during OA.

## Results

### Degeneration Model of Articular Cartilage

**Figure 1:**
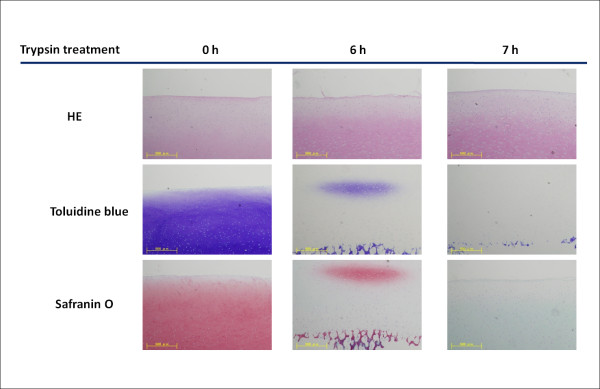
**Results of histological examination**. After treatment for 7 h, no staining was detected in samples with toluidine blue and safranin O stains, but only HE staining. Therefore, cartilage plugs treated for >7 h were considered as degenerated cartilage models in these experiments. Scale bar = 500 μm.

Figure 1 shows the histological appearance of trypsin-treated cartilage samples. As demonstrated by the staining results for porcine articular cartilage using HE, toluidine blue and safranin O, normal cartilage tissue changed into degenerated tissue over time and staining patterns altered after enzyme treatment. At the starting point of enzyme digestion (0 h), cartilage samples showed uniform staining throughout with toluidine blue and safranin O. However, increasing degeneration resulted in larger loss of such staining over time, showing extensive loss of proteogylcans in the tissue.

After 7 h of digestion, no staining was detected in samples using toluidine blue or safranin O, with only HE staining remaining. Therefore, treating cartilage plugs for >7 h was considered to achieve suitable models of degenerated cartilage in these experiments. When 6 h had passed, center regions of digested samples still showed a small portion of intensive staining with safranin O and metachromatic staining with toluidine blue, demonstrating that normal ECM constituents are still present, unlike samples treated for >7 h in which ECM protein components have totally disappeared. Degenerated cartilage and untreated normal cartilage samples were used in FCS measurements.

### FCS Measurements

**Figure 2:**
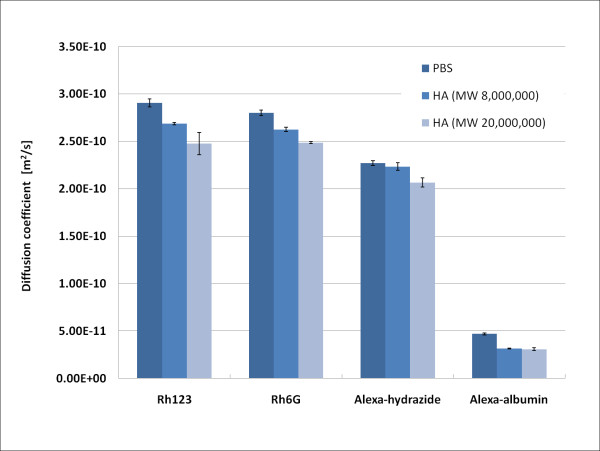
**Diffusion coefficients of fluorescent dyes, E-10 = ×10^-10^**. Diffusion coefficients of fluorescent dyes were in the order of PBS > HA (MW, 8.0 ×10^5^) > HA (MW, 2.0 ×10^6^).

Diffusional behaviors (diffusion coefficient) for all fluorescent dyes utilized in this research were detected by FCS monitoring in PBS solvent. Optimal concentrations of fluorescent dyes differed, which may have resulted from the different chemical, properties of dyes in solusions. The optimal concentrations of Rhodamine 123, Rhodamine 6G, Alexa Fluor 488 hydrazide, and Alexa Fluor 488 conjugated with albumin form bovine serum were, 10^-7^M, 10^-7^M, 10^-8^M, 10^-5^M, respectively. Two different HA, Artz (MW; 8.0 ×10^5^, Seikagaku, Tokyo, Japan) and Suvenly (MW; 2.0 ×10^6^, Chugai Pharmaceutical, Tokyo, Japan) were used and their concentrations were 0.1 wt%. Diffusion coefficients of the fluorescent dyes were in the order of PBS > HA (MW, 8.0 ×10^5^) > HA (MW, 2.0 ×10^6^) (Figure. 2).

Changes of diffusion coefficients are related to the MW of dyes and apparent viscosity of aqueous solution. Fluorescent dyes with a larger MW showed lower diffusion coefficients. At the same time, diffusion coefficients of probe dyes in solution containing HAs decreased with increasing MW of HAs.

The fluorescent dye validated in HAs and cartilage tissues was accepted as a FCS probe, and then applied in the next tests. Since the maximum measurable depth for FCS equipment using Alexa dyes was greater than that using Rhodamine dyes (data not shown), Alexa Fluor 488 was selected as the FCS probe.

**Figure 3:**
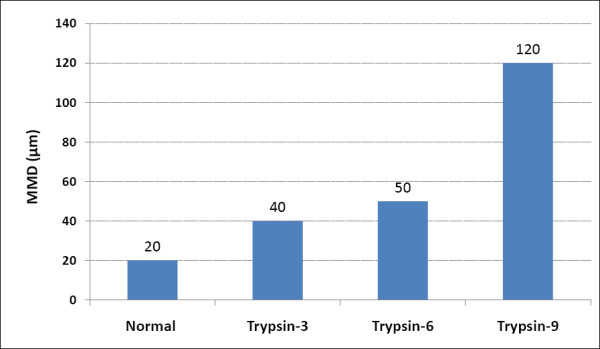
**Maximum measurable depth (MMD)**. FCS data demonstrated an intimate correlation between measureable points, MMD, and prolonging the reaction time of trypsin treatment.

FCS measurement tests were performed with Alexa Fluor 488 labled-albumin to trace the diffusion motion of albumin in both normal and degenerated cartilage. FCS data demonstrated an intimate correlation between measureable points (depths from the superficial surface) and enzyme-treatment times (Figure. 3). Increments in these points were correlated with prolongation of trypsin treatment times. FCS data were validated at the range of 120 μm when digestion was conducted for 9 h and >9 h, showing that FCS monitored permeation of the FCS probe at this depth. These focus distances were defined as maximum measurable depths (MMD). When the focus moved over these ranges, no correlation curves were formed, indicating that no movement and no localization of fluorophores is detected in the testing field. As a result of MMD detection, an MMD of 20 μm was chosen for cartilage tissue in the present study.

**Figure 4:**
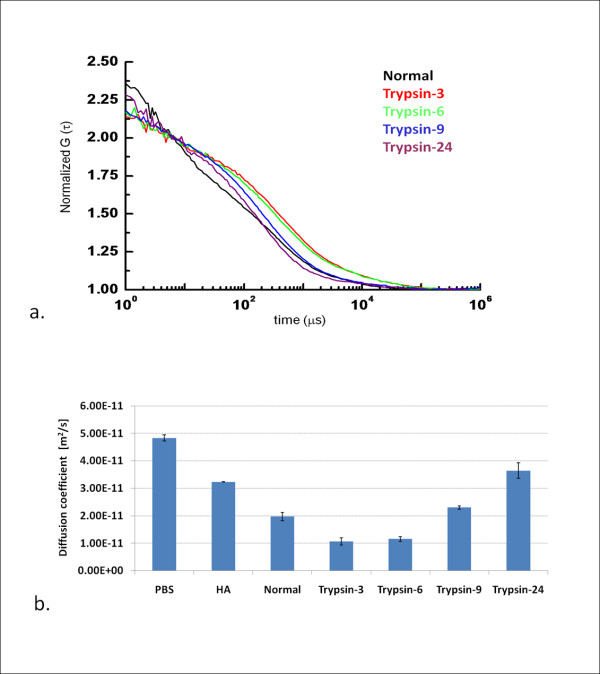
**Summary of the FCS measurements**. a. Representative of auto correlation curves for Normal, Trypsin-3, Trypsin-6, Trypsin-9, and trypsin-24 b. Diffusion coefficients of Alexa-albumin, E-10 = ×10^-10^. FCS data demonstrated that diffusion coefficients of Alexa Fluor 488 conjugated with albumin (Alexa-albumin) were greater when measuring degenerated cartilage, indicating that FCS probes moved more freely in degenerated cartilage than in normal tissue. Interestingly, diffusion coefficients for trypsin-3 and trypsin-6 were lower than in normal cartilage. *p *values of each of groups were under 0.01 which demonstrated the significance of the difference between the groups excluding three of *p *values between the groups; HA vs.Trypsin-24, Normal vs. Trypsin-9, and Trypsin-3 vs. Trypsin-6. Among these higher values over 0.01, *p *value between No significant difference was found between Trypsin-3 and Trypsin-6(*p *= 0.486). Besides, we found a significant difference in the rest of two values which were below 0.05 (*p *= 0.042; between HA and Trypsin-24, and *p *= 0.021; Normal and Trypsin-9).

FCS measurement data for Alexa Fluor 488 conjugated with albumin from bovine serum (Alexa-albumin) as the FCS probe are summarized in Figure 4, which explains the diffusion behaviors of albumin under varying circumstances. We selected one HA with an MW of 8.0 ×10^5 ^as representative and performed analyses for this HA. Diffusion coefficients of Alexa-albumin in PBS, HA, trypsin-treated cartilage for 3 h (Trypsin-3), trypsin-treated cartilage for 6 h (Trypsin-6), trypsin-treated cartilage for 9 h (Trypsin-9), and trypsin-treated cartilage for 24 h (Trypsin-24), were 4.83 ×10^-11^, 3.23 ×10^-11^, 1.06 ×10^-11^, 1.15 ×10^-11^, 2.30 ×10^-11^, and 3.64 ×10^-11 ^m^2^/s, respectively. In addition, non-treated normal cartilage was 1.97 ×10^-11^, between the ranges of Trypsin-6 and Trypsin-9. An increase in diffusion coefficients was seen with increased duration of chemical digestion.

*p *values of each of groups were under 0.01 which demonstrated the significance of the difference between the groups excluding three of *p *values between the groups; HA vs.Trypsin-24, Normal vs. Trypsin-9, and Trypsin-3 vs. Trypsin-6.

## Discussion

FCS is an extremely sensitive method for providing concentration and diffusion constant, and molecular interaction of fluorescent molecules in a small volume (femtoliters) of complex mixtures [1-3]. The major sources of fluctuations within the confocal volume are molecular diffusion by Brownian motion, convection, and chemical reactions that change the fluorescence yield. The parameters of molecular dynamics can then be easily extracted by analyzing the time correlation function of fluorescence fluctuations. Information from FCS monitoring is expressed by the autocorrelation curve, then diffusion behaviors are analyzed for comparison. Recent research has been extensively applying FCS measurements to material transportation and interrelationships among biomolecules at the level of the single living cell, and even at the ultra-micro level of intracellular spaces in small organelles [3,9,10].

One important cartilage proteoglycan is HA (comprising glucuronic acid and N-acetyl-glucosamine). This molecule is one of the major components in synovial fluid. HA molecules are also present in cartilage matrix as the backbone structure in proteoglycan aggregates. Since HA plays a major role as an organizer of the ECM [11], we selected HA as a test solution for a FCS probe of cartilage tissue in addition to PBS as a solvent.

In the present study, we tried to measure diffusion properties in cartilage tissues using FCS methods. Before this step, a suitable FCS probe was needed to trace the motion of albumin, and preliminary investigation of several fluorescence dyes was conducted to assess their properties. All diffusion characters of the fluorescent dyes in the present research were detected by FCS equipment in the solvent of PBS and HAs. Since rhodamine dyes showed unstable when applied to cartilage tissue, Alexa dyes utilized as FCS probe. This instability may concern several reasons of character of rhodamine itself such as its strong absorption, tendency to dimerize at higher concentration[12] as so on. Further investigation is needed to determine most proper dye when fluorescent dye is applied to other tissue.

OA is caused by alterations in proteoglycans, degeneration of the collagen network, and an increase in fluid content [13]. Experimental treatments using specific enzymes such as trypsin can simulate these changes [14,15]. In this study, we chose cartilage degeneration created using short-term trypsin treatment as a model of OA. Trypsin treatment of tissue caused a marked loss of proteoglycans in the cartilagenous tissue (Figure. 1). Degenerated cartilage was defined with treatment for >7 h, showing no staining with toluidine blue or safranin O. This result is consistent with previous findings [15].

FCS data demonstrated that diffusion coefficients of Alexa-albumin were greater when measuring degenerated cartilage (Figure. 4), indicating that FCS probes moved more freely in degenerated cartilage than in normal tissue. This phenomenon may occur due to the gradual destruction of ECM structures, finally giving probes a chance to move in a wider space than that in normal cartilage. Diffusion coefficients provide diffusion characteristics of certain molecules. Molecules with larger MW showed lower diffusion coefficients (Figure. 2). Diffusivity decrease with increasing molecular size of flurorescent dyes. Simultaneously, specific molecule described by the diffusion coefficient is affected by the solution environment as shown by tests of HAs showing decreasing diffusion coefficients with increasing MW. Therefore, lower diffusion coefficients indicate difficulties for molecules to diffuse within the surrounding conditions, matching the present results. We were therefore satisfied that the diffusion coefficient for Alexa-albumin can explain the diffusion characteristics in PBS, HAs and cartilage tissue. Interestingly, diffusion coefficients for trypsin-3 and trypsin-6 were lower than in normal cartilage. Considering parameters of diffusion equations and our findings from this study together, this result may be explained as follows. When an aggressive chemically digestion using trypsin induced cartilage degeneration is initiated, early-stage ECM denaturation may change the natural space of the ECM to much more intricate surroundings with exudates digested ECM constituents that hinder albumin diffusion and even have possibility of non-specific interaction with albumin. Moreover, this finding may reflect the time-related degeneration of cartilage, which of early stage differs from that of late stage. Once cartilage has been treated for >6 h, albumin is more diffusible and thus shows an increased diffusion coefficient compared to normal cartilage.

Synovial fluid is a lubricant of intra-articular surfaces and a source of nutrients for hyaline cartilage by diffusion. Synovial protein concentration averages around 42% of the concentration in serum [16]. Among these proteins, albumin constitutes the single largest protein fraction (55-86% of total synovial protein) [16], representing a major contribution to the role of colloid osmotic pressure and other physiobiological functions. As a result, understanding the diffusion characteristics of albumin in such synovial fluid is valuable. Alexa-albumin is made by adding labeled albumin from bovine serum to Alexa-hydrazide. The diffusion behavior of this fluorescent dye was reproducible and simulated experimentally with an *in vitro *model tracing the movement of albumin. Alexa-albumin happened to be commercially available, but FCS probes using other molecules will be also feasible as candidates to create a standard for understanding physiological diffusion in the living body. In addition to albumin as a target molecule for FCS probes, further studies regarding new FCS molecular probes will be essential in the future. The present results reveal that the diffusion state of fluorescence-dyed albumin can be determined by FCS measurement regardless of the intensity of cartilage degeneration.

Some studies have already examined diffusion of particular dyes in cartilage tissue [6-8]. However, methods in those experiments were unsuitable for application as diagnostic tools. These studies needed large volumes of cartilage tissue and were based on macroscopic (gross) experimental data, representing an extremely high level of invasiveness. One of the experiments with dyes and glucose was utilized to evaluate the mechanisms of diffusion across the cartilaginous membrane in a two-compartment device [6]. To diagnosis the pathological alterations in cartilage clinically using diffusion information, simple preparation of samples for examination is critical for achieving minimally invasive diagnosis. In a similar trial in terms of using fluorescence materials and tiny detection fields, Hardingham et al. developed sensitive methods for assessing the matrix assembly around chondrocytes, based on the use of confocal fluorescence recovery after photobleaching (confocal-FRAP) to determine the translational diffusion of fluorescent tracer molecules of defined size [17]. However, this technology was not devised to measure diffusion of particular molecules directly in the ECM, but rather to elucidate the conditions for matrix assembly itself.

More recently, studies have been reported and they showed different methods of measuring diffusion in cartilage using different fluorescence techniques [18-28]. Histological evaluations were conducted on tidemark and calcified cartilage of histological sections with fluorescence agents (fluorecein and rhodamine) [24] itself, and fatty acid labeled with rhodamine fluorescence and albumin[25] using quantitative fluoresce microscopy.

Several researchers reported photobleaching methods pioneered with fluorescence recovery after photobleaching (FLAP) [18,26], fluorescence loss induced by photobleaching (FLIP) [19] and scanning microphotolysis (SCAMP) [20-23]. Fluorecein [18,19], fluorescently-labeled dextran(FITC-conjugated dextran) [20-23] and fluorecein-conjugated bovine serum albumin [26] were utilized as a fluorescent materials to evaluate the diffusivity of human annulus fibrosus [18], calcified cartilage of deep region [19], pericellular matrix of porcine articular cartilage [21], cartilage in normal state [20,23,27,28] or during compression with mechanical stress [22,27,28], ligament [23], growth plate [28] and even agarose [26] and tissue-engineered cartilage [20]. These studies have reported the diffusive transport properties of solutes in both cartilage and collagenous tissue. Some of the experimental results for diffusion coefficients, determined by various methods using fluorescent probes, are summarized in Table 1. According to this table, diffusion coefficient (*D*) ranged from 2.0 ×10^-14 ^to 290.0 ×10^-10 ^m^2^/s. Nonetheless, most of numbers for diffusion coefficients measured with different fluorescent dyes are of similar magnitude to those that measured with FCS in our experiment, on the order of 10^-11 ^to 10^-10^. Our adaptation of the FCS technique can be used to measure site-specific diffusivity in an extremely small detection volume of tissue. The diffusion coefficients measured with Alexa-albumin in the normal cartilage (1.97 ×10^-11 ^m^2^/s) and degenerated cartilage (1.06 ×10^-11^, 1.15 ×10^-11^, 2.30 ×10^-11^, and 3.64 ×10^-11 ^m^2^/s), are in good agreement with values of the order measured previously using other techniques (3.1 ×10^-11 ^m^2^/s using fluorescence recovery after photobleaching and 4.0 ×10^-11 ^m^2^/s using radiotracertracking [21]. Among these previous studies with tagged with albumin probes [25,26], the diffusion coefficients in cartilage ranged from 0.3 ×10^-11 ^to 29.0 ×10^-11 ^m^2^/s (Table 1). Considering the range of methods and possible variation in the properties of various cartilage sources, present results (Figure. 4) are in reasonable agreement with these data, supporting the accuracy of our methods. Advantages of our methods include quickness of diffusion measurements, simplicity, noninvasiveness, and the ability to quantify the molecular diffusion in the different individual tissues.

**Table 1:** Summary of experimental results for diffusion coefficient, *D*, from recent studies using fluorescent dye.

Fluorescent dye	Method	Specimen	Temp. (°C)	***D *(**×**10**^**-10**^**m**^**2**^**/s)**	**Ref**.
Fluorecein (332 Da)	Fluorescence recovery after photobleaching (FRAP)	Human intervertebral discs	22	0.38 ± 0.25 ~ 2.68 ± 0.84	[18]
		Inner, middle and outer regions of annulus fibrosus			

Fluorecein (376 Da)	Fluorescence loss induced by photobleaching (FLIP).	Murine (C57BL6J) distal humurs	4		[19]
		Subchondral bone		0.0002 ~ 0.012 (0.0007 ± 0.0003)	
		Calcified cartilage		0.0005 ~ 0.009 (0.0026 ± 0.0022)	

Fluorescein isothiocyante (FITC)-tagged dextran (3, 40, 70, and 500 kDa)	Fluorescence recovery after photobleaching (FRAP)	Tissue engineered cartilage from human adipose-derived stem cell with or without scaffold (alginate, agarose, fibrin and gelatin)	37	0.16 ± 0.08 (Day 28, cultured within fibrin in control media using 500 kDa)	[20]
			or	~ 18.10 ± 3.94 (Day 1, cultured within gelatine in chondrogenic media using 3 kDa)	
			?		

Fluorescein isothiocyante (FITC)-tagged dextran (70 kDa)	Scanning microphotolysis (SCAMP).	Porcine femoral condyle	?		[21]
		Healthy cartilage			
		Extracellular matrix		0.23 ± 0.02	
		Pericellular matrix		0.19 ± 0.02	
		Osteoarthritic cartilage			
		Extracellular matrix		0.23 ± 0.02	
		Pericellular matrix		0.23 ± 0.02	

Fluorescein isothiocyante (FITC)-tagged dextran (70 kDa)	Scanning microphotolysis (SCAMP) and Fluorescence imaging of continuous point photobleaching (FICOPP)	Porcine femoral condyle	?		[22]
		Normal cartilage		0.33	
		Compressed cartilage		0.07	

Fluorescein isothiocyante (FITC)-tagged dextran (3 and 500 kDa)	Fluorescence imaging of continuous point photobleaching (FICOPP)	Collagenous tissues	4	Inexpressible because authors explain diffusivity by not diffusion coefficient but by diffusivity ratio for comparisons	[23]
		3% agarose gel	or		
		Lateral collateral ligaments (Porcine)	?		

Rhodamine B (443 Da, cationic), Rhodamine B (479 Da, neutral but polar), Fluorecein (332 Da), and Na-fluorecein (376 Da)	Quantitative fluorescence microscopy on histological sections	Equine forelimb	4	0.0098 ± 0.0013 ~ 0.037 ± 0.003	[24]
		Subchondral bone			
		Calcified cartilage			

Bovine serum albumin labeled with rhodamine - maleimide and Nitrobenz -2-oxa-1,3-diazole (NBD) -labelled lauric acid (378 Da) bound to the fluorescent albumin	Quantitative fluorescence microscopy on histological sections	Equine metacarpal-phalangeal joints	4	9.0 ± 2.0 (48 h-incubation, using albumin)	[25]
				~	
				290.0 ± 10.0 (2 h-incubation, using lauric acid)	

Fluorescein-conjugated bovine serum albumin (66 kDa).	Fluorescence recovery after photobleaching (FRAP)	3~8% agarose gel	24	0.164 ± 0.018 ~ 0.411 ± 0.008	[26]
		Porcine growth plate		0.0387 ~ 0.4922	

Tetramethylrhodamine (TMR) -tagged dextran (3,10, and 40 kDa) and tetramethylrhodamine (430 Da) itself,	Novel experimental apparatus and desorption fluorescence method.	Bovine femurs	4	0.19 ± 0.02 (8% compression, using 40 kDa dextran)	[27,28]
		Compressed cartilage		~	
				0.52 ± 0.06 (8% compression, using 430 Da TMR)	

For studies investigating anisotropic diffusivity, the smallest to the largest value of the diffusion coefficient is reported. (Temp.: Temperature, Ref.: Reference)

The apparently wide range of diffusivities of normal cartilage and degenerated cartilage highlights the influence of physical properties of both the fluorescent molecule and the ECM on hindered transport within biological systems. This may results from the circumstantial and collateral conditions such as tissue conditions (animal species, type of cartilage, preservation until measurement, compression etc.), solutions utilized (ingredient, ion contents, buffer, culture media, manufacturing company etc.), tissue processing (treatment and incubation time, condition, time, temperature etc.), and properties of each fluorescent dye (shape, molecular weight, physical properties electric charge, the hydrophilic or hydrophobic natures, manufacturing company ect).

Diffusion coefficients can also be measured by fluorescence recovery after photobleaching (FRAP). However, most of these methods to analyze FRAP data expect the homogeneity in the measurable field of the bleached area and fail to assume geometrical restrictions to diffusion. Accordingly, diffusion coefficients in inhomogeneous materials, such as most biological tissues, cannot be evaluated correctly.

Several methods are available to analyze FRAP data, each with its own characteristics [29]. The technique to apply depends on the data which are aimed for and the tissue that is being probed. Presumably the most adaptable tool currently utilized is by spatial Fourier analysis of a sequence of FRAP images [30]. With this method, anisotropic diffusion, flow, matrix binding, and diffusivity in multiple components of a gel can be evaluated, whereas the evaluation is independent on the geometry of the bleached area [26]. This limitation originates from the requirement that the boundary of the image must have a constant intensity value. In practice, this means that a large area, relative to the bleached area, is to be imaged. This decreases the amount of signal in the images. The same requirement of constant boundary intensity applies to this method [26]. The average intensity of the images is allowed to change during the measurements. In practice, this means that the bleached area typically constitutes a large part of the acquired images to enhance the signal. Note that the lower limit to the physical size of the bleached area is defined by the point-spread function. This needs to be considered if small bleached areas are used [26].

Compared with photobleaching methods, FCS need minimum excitation power. Hence, this technique requires lower power and much small amount of fluorescent dyes to get information of FCS data. It is not easy to calculate directly the diffusion coefficient with photobleaching tools, however, we can promptly get the absolute value of diffusion coefficient with FCS instrument. One of the merits using FCS is that various concentrations of the fluorescent molecules used are easily monitored. It is worth considering the complementary use of FCS and photobleaching methods with their different characteristics.

To the best of our knowledge, no previous studies have demonstrated the feasibility of FCS for direct measurement of diffusional behaviors of fluorescently labeled albumin in the ECM of cartilage tissue, and this approach may represent a potential and useful evaluation tool. If diffusion in the ECM can be clarified and categorized with this new method by standardization of FCS data under various cartilage conditions, FCS will be applicable for the early diagnosis of OA, which is accompanied by increased destruction of ECM elements, and also as a tool for evaluating bio-engineered artificial cartilage for autologous chondrocyte implantation. Besides changes in diffusion characteristics of molecules in the cartilage ECM, additional and complementary information can be adopted to clarify the clinical picture. For example, alterations in the ECM simultaneously induce changes in viscoelasticity of the cartilage. Monitoring changes in viscoelasticity is possible using reliable techniques such as photoacoustic measurement [15,31,32]. Such information could be used together with diffusion characteristics to evaluate optimal conditions for ECM and to test bio-engineered neocartilage constructs, and will suggest new criteria for real-time evaluation with small quantities of samples under minimally invasive arthroscopic surgery with FCS analysis system.

## Conclusions

This work offers the first demonstration of the capabilities of FCS for direct measurement of diffusion behaviors of ECM in cartilage. This sensitive measurement technique provides great advantages in detecting diffusible molecules due to the ability to achieve rapid measurements from small sample volumes.

## Methods

### Sample Preparation

Fresh swine knees (n = 5) were obtained from a local slaughterhouse at Kanagawa meat center (Frieden, Kanagawa, Japan). Cartilage tissue from the femur was prepared within 4 h as follows. The cartilage tissues of delivered knee joints were cut out into cylindrical cartilage plugs (diameter, 5 mm; depth, 1 mm; n = 240) using a biopsy punch (Kai Industries, Seki City, Japan) and disposable scalpels (Akiyama, Tokyo, Japan). Porcine cartilage specimens were initially incubated under physiological conditions (37°C, 5% CO_2_) in physiological saline (Otsuka Pharmaceutical Factory, Tokushima, Japan) until the next procedure.

### Degenerated Cartilage Models

Addition of enzymes was used for experimental degradation of the tissue matrix using phosphate-buffered saline (PBS) (Wako Pure Chemical, Osaka, Japan) containing 0.1% trypsin solution (1 mg/ml; Invitrogen, Carlsbad, CA, USA) to degrade primarily proteoglycans. The trypsin treatment time was minimally 1 h and varied up to 24 h every hour to control the extent of degeneration.

To stop the trypsin reaction, an equal volume of fetal bovine serum (FBS) (Invitrogen) was added and then incubated for a further 30 min. The digested cartilage samples were thoroughly rinsed with PBS to remove residual trypsin and FBS.

Chemically treated and non-treated cartilage discs were then divided for histopathological assessment and FCS measurement. To perform the histopathological assessment, samples were fixed in 4% paraformaldehyde and embedded in paraffin, and 4-mm-thick sections were prepared. Histological staining was performed using hematoxylin and eosin (HE), toluidine blue and safranin O to visualize the degree of ECM degeneration within specimens. Samples for FSC were prepared and used for FCS measurements with the FCS probes as described below.

### Preparation and Selection of Fluorescent Dye

To determine properly applicable fluorescent dyes (FCS probes) for cartilage tissue, commercially available fluorescent dyes were obtained, including Rhodamine 123 (Rh123) (molecular weight (MW), 380.82; Sigma-Aldrich, St. Louis, MO) Rhodamine 6G (Rh6G) (MW, 479.01; Sigma-Aldrich), Alexa Fluor 488 hydrazide (Alexa-hydrazide) (MW, 570.48; Molecular Probes, Eugene, OR). First, optimal concentrations of the fluorescent dyes in PBS (100 μl) were measured and analyzed, then applicable concentrations of each dye solution (100 μl) were mixed with 100 μl of hyaluronic acid sodium (HA) and tested.

To optimalize the concentrations of these fluorescent probes, FCS measurement were conducted with ten-fold serial dilutions of each fluorescent agents adding PBS. After receiving the FCS data from these measurements, the calculatable data of each concentration of dyes were obtained by expressing autocorrelation curve.

Two different molecular sizes of HA with average MWs of 8.0 ×10^5 ^(Artz; Seikagaku, Tokyo, Japan) and 2.00 ×10^6 ^(Suvenly; Chugai Pharmaceutical, Tokyo, Japan) were used in this experiment.

In addition to these measurements, albumin-conjugated fluorescent dyes were adopted to evaluate the diffusion behavior of albumin protein. Alexa Fluor 488 conjugated with albumin from bovine serum (Alexa-albumin) (MW approximately 66,000; Molecular Probes) was tested and analyzed with PBS, two differ types of HA solutions and cartilage samples. Physiological parameters affecting the diffusion behaviors of fluorescence dyes were measured and monitored with FSC measurement, including counts per molecule, count rate, diffusion time, particle number, correlation, structure parameter, triplet fraction and triplet time. In the case of measurement within cartilage tissues, length from the superficial surface to the maximum measureable points was experimentally determined.

### Statistical Analysis

All results of the experiments are expressed as the means ± SE. The mean values for each group were compared by ANOVA and then by using Fisher's least significant difference method. Values of *p *< 0.05 were considered the minimum level of statistical significance

### FCS Measurement and Analysis

FCS was performed using an LSM510-ConfoCor 2 system (Carl Zeiss, Oberkochen, Germany), as described elsewhere [33,34]. FCS measurements of all samples were recorded at 25°C.

Various concentrations of the candidate fluorescent dyes were incubated with purified PBS at 37°C in an atmosphere of 5% CO_2 _and 95% air over 30 min. Aliquots (100 μl) were arrayed onto Lab-Tek chambered cover-glass (Nalge Nunc International, Naperville, IL, USA) with eight wells and <140-μm-thick cover-glass on the bottom. Cartilage specimens were placed on Lab-Tek chambered cover-glass with eight wells and 100 μl of FCS probe-solution was applied over the samples.

Each acquired correlation data set was analyzed by software supplied by Carl Zeiss with a fitting program (FCS Access Fit software; EVOTEC BioSystems, Hamburg, Germany), or exported to Igor Pro software (IGOR Pro 5.05a; Wavemetrics, Lake Oswego, OR). In the FCS analysis, the diffusion coefficient is represented by the average of five FCS measurements

The autocorrelation curve is obtained by correlating the fluorescence intensity trace shifting within a time interval. The time shift *τ *is varied, and the correlation curve is obtained by multiplying the deviation of the average intensity, *δF*, at the time point *t *with the deviation at time point *t *+ *τ *and averaging over the whole trace. Finally, the correlation function, *G(τ)*, is normalized with the squared average signal.

(1) $$G\left(\tau \right)=\frac{\langle \delta F(t)\delta F\left(t+\tau \right)\rangle }{{\langle F\rangle }^{2}}$$

Further practical considerations in the calculation of FCS curves from a fluorescence intensity trace are detailed elsewhere [35-37].

Diffusion of one single component is commonly fitted with the standard model [38]:

(2) $$G\left(\tau \right)=1+\frac{1}{N}{\left(1+\frac{4D\tau }{\omega_{0}{}^{2}}\right)}^{-1}{\left(1+\frac{4D\tau }{z_{0}{}^{2}}\right)}^{-\frac{1}{2}}$$

The resulting ideal probe volume is approximated by a Gaussian profile with the extension ω_0 _in x and y directions and *z*_*0 *_in the z direction [39]. *N *is the number of fluorescence molecules in the detection volume, defined by a radius ω_0 _and a length 2 z_0_. The diffusion time (τ_*D*_) is related to the traditional diffusion constant of the diffusion coefficient *D*. As this time corresponds to Equation 3, the diffusion coefficient *D *is obtained:

(3) $$\tau_{D}=\frac{\omega_{0}{}^{2}}{4D}$$

The diffusion of spherical molecules is related to various physical parameters by the Stokes-Einstain equation as follows:

(4) $$D=\frac{k_{B}T}{6\pi \eta r}$$

where *T *is the absolute temperature, *r *is the radius of the spherical molecule, *η *is the fluid-phase viscosity of the solvent, and *k*_*B *_is the Boltzman constant.

When measuring the diffusion time of samples (τ_sample_) and rhodamine 6G (τ_Rh6G_) with the FSC system, the diffusion coefficient of rhodamine 6G at 20°C [40], 2.8 ×10^-10^m^2^/s, was used as an authentic value for determination of the diffusion coefficient of samples (*D*_*sample*_) measured on the expectation of a proportional relationship based on the following equation:

(5) $$\frac{D_{sample}}{D_{Rh6G}}=\frac{\tau_{Rh6G}}{\tau_{sample}}$$

## Competing interests

The authors declare that they have no competing interests.

## Authors' contributions

JIL and KU performed the research. JIL, KU and MS analyzed the data. JIL took charge of the statistical analyses. JIL, KU, MS, and JM wrote the manuscript. All authors have read and approved the final manuscript.
